# Supplementary material for: Knowledge, Beliefs, Dietary, and Lifestyle Practices Related to Bone Health among Middle-Aged and Elderly Chinese in Klang Valley, Malaysia
Source: Int J Environ Res Public Health. 2019 May 20;16(10):1787. doi: 10.3390/ijerph16101787 (PMC6572024; doi:10.3390/ijerph16101787)
Supplement: Supplementary file 1 [file ijerph-16-01787-s001.pdf]

Supplementary Materials

Table S1. The distribution of responses to osteoporosis knowledge questions of subjects.

Items	Correct Ans	Overall (N = 367)			Men (n = 182)			Women (n = 185)			Correct Responses (N = 367)	
		T	F	DK	T	F	DK	T	F	DK	n	%
1. Makes bone become brittle and weak from loss of tissue, thus more likely to break (fracture).	T	353 (96.2)	11 (3.0)	3 (0.8)	174 (95.6)	6 (3.3)	2 (1.1)	179 (96.8)	5 (2.7)	1 (0.5)	353	96.2
2. Osteoporosis will result in knee pain.	F	217 (59.1)	91 (24.8)	59 (16.1)	103 (56.6)	45 (24.7)	34 (18.7)	114 (61.6)	46 (24.9)	25 (13.5)	91	59.1
3. Postmenopausal women have no risk to develop osteoporosis.	F	14 (3.8)	284 (77.4)	69 (18.8)	11 (6.0)	113 (62.1)	58 (31.9)	3 (1.6)	171 (92.4)	11 (5.9)	284	77.4
4. Osteoporosis is an untreatable disease.	F	90 (24.5)	223 (60.8)	54 (14.7)	40 (22.0)	113 (62.1)	29 (15.9)	50 (27.0)	110 (59.5)	25 (13.5)	223	60.8
5. Osteoporosis can be diagnosed by measuring bone mineral density.	T	337 (91.8)	6 (1.6)	14 (6.5)	165 (90.7)	2 (1.1)	15 (8.2)	172 (93.0)	4 (2.2)	9 (4.9)	337	91.8
6. I do not need to do bone mineral density test unless I fracture my bones.	F	33 (9.0)	328 (89.4)	6 (1.6)	21 (11.5)	156 (85.7)	5 (2.7)	12 (6.5)	172 (93.0)	1 (0.5)	328	89.4
7. Consume calcium supplements help me to prevent osteoporosis.	T	300 (81.7)	40 (10.9)	27 (7.4)	150 (82.4)	19 (10.4)	13 (7.1)	150 (81.1)	21 (11.4)	14 (7.6)	300	81.7
8. The regular intake of calcium supplements can lead to formation of kidney stones.	F	208 (56.7)	54 (14.7)	105 (28.6)	101 (55.5)	24 (13.2)	57 (31.3)	107 (57.8)	30 (16.2)	48 (25.9)	54	14.7
9. Foods such as milk, tofu, yellow dhal and spinach are rich in calcium.	T	342 (93.2)	8 (2.2)	17 (4.6)	162 (89.0)	6 (3.3)	14 (7.7)	180 (97.3)	2 (1.1)	3 (1.6)	342	93.2
10. By exposing my skin to sunlight for about 15 minutes a day, I can obtain my recommended daily intake of vitamin D.	T	214 (58.3)	108 (29.4)	45(12.3)	117 (64.3)	46 (25.3)	19 (10.4)	97 (52.4)	62 (33.5)	26 (14.1)	214	58.3
11. Intake of glucocorticoids may increase the risk of osteoporosis.	T	122 (33.2)	28 (7.6)	217 (59.1)	61 (33.5)	9 (4.9)	112 (61.5)	61 (33.0)	19 (10.3)	105 (56.8)	122	33.2
12. I must have good vision and comfortable shoes with good grip to prevent me from falling.	T	328 (89.4)	36 (9.8)	3 (0.8)	164 (90.1)	17 99.3)	1 (0.5)	164 (88.6)	19 (10.3)	2 (1.1)	328	89.4

* *T:* True, *F:* False, *DK:* Don't Know.

Table S2. Response to the OHBS among the subjects.

Items	Health Beliefs Scale, N (%)														
	Overall (N = 367)					Men (n = 182)					Women (n = 185)				
	SD	D	N	A	SA	SD	D	N	A	SA	SD	D	N	A	SA
1. You have high chance to get osteoporosis.	4 (1.1)	97 (26.4)	92 (25.1)	155 (42.2)	19 (5.2)	3 (1.6)	63 (34.6)	46 (25.3)	63 (34.6)	7 (3.8)	1 (0.5)	34 (18.4)	46 (24.9)	92 (49.7)	12 (6.5)
2. You more likely to get osteoporosis because of your family history.	7 (1.9)	223 (60.8)	51 (13.9)	78 (21.3)	8 (2.2)	4 (2.2)	112 (61.5)	20 (11.0)	42 (23.1)	4 (2.2)	3 (1.6)	111 (60.0)	31 (16.8)	36 (19.5)	4 (2.2)
3. It would be very serious if you got osteoporosis.	1 (0.3)	65 (17.7)	47 (12.8)	207 (56.4)	47 (12.8)	1 (0.5)	29 (15.9)	26 (14.3)	98 (53.8)	28 (15.4)	-	36 (19.5)	21 (11.4)	109 (58.9)	19 (10.3)
4. Exercise regularly helps to build strong bones.	1 (0.3)	17 (4.6)	14 (3.8)	275 (74.9)	60 (16.3)	-	7 (3.8)	10 (5.5)	127 (69.8)	38 (20.9)	1 (0.5)	10 (5.4)	4 (2.2)	148 (80.0)	22 (11.9)
5. Intake of enough calcium prevents you from getting osteoporosis.	-	31 (8.4)	39 (10.6)	273 (74.4)	24 (6.5)	-	16 (8.8)	19 (10.4)	135 (74.2)	12 (6.6)	-	15 (8.1)	20 (10.8)	138 (74.6)	12 (6.5)
6. You feel that you are not strong enough to exercise regularly.	21 (5.7)	233 (63.5)	36 (9.8)	74 (20.2)	3 (0.8)	18 (9.9)	129 (70.9)	9 (4.9)	24 (13.2)	2 (1.1)	3 (1.6)	104 (56.2)	27 (14.6)	50 (27.0)	1 (0.5)
7. Starting a new habit to exercise regularly is hard for you to do.	16 (4.4)	249 (67.8)	43 (11.7)	58 (15.8)	1 (0.3)	14 (7.7)	135 (74.2)	12 (6.6)	20 (11.0)	1 (0.5)	2 (1.1)	114 (61.6)	31 (16.8)	38 (20.5)	-
8. Calcium-rich foods are very costly.	6 (1.6)	297 (80.9)	36 (9.8)	26 (7.1)	2 (0.5)	2 (1.1)	147 (80.8)	21 (11.5)	11 (6.0)	1 (0.5)	4 (2.2)	150 (81.1)	15 (8.1)	15 (8.1)	1 (0.5)
9. You do not like calcium-rich foods.	9 (2.5)	307 (83.7)	42 (11.4)	9 (2.5)	-	5 (2.7)	146 (80.2)	26 (14.3)	5 (2.7)	-	4 (2.2)	161 (87.0)	16 (8.6)	4 (2.2)	-
10. You always look for new information related to health.	-	32 (8.7)	65 (17.7)	255 (69.5)	15 (4.1)	-	20 (11.0)	30 (16.5)	125 (68.7)	7 (3.8)	-	12 (6.5)	35 (18.9)	130 (70.3)	8 (4.3)
11. Even you are not sick, but you will do regular health check-up.	-	82 (22.3)	35 (9.5)	239 (65.1)	11 (3.0)	-	40 (22.0)	15 (8.2)	123 (676)	4 (2.2)	-	42 (22.7)	20 (10.8)	116 (62.7)	7 (3.8)
12. You will follow recommendations to keep you healthy.	1 (0.3)	2 (0.5)	39 (10.6)	308 (83.9)	17 (4.6)	-	1 (0.5)	16 (8.8)	155 (85.2)	10 (5.5)	1 (0.5)	1 (0.5)	23 (12.4)	153 (82.7)	7 (3.8)

* *SD:* Strongly Agree, *D:* Disagree, *N:* Neutral, *A:* Agree, *SA:* Strongly Agree.
